# Excreting the stress: a novel regulatory cascade drives salt gland development in *Limonium bicolor*

**DOI:** 10.1093/plphys/kiag305

**Published:** 2026-05-23

**Authors:** Guannan Wang

**Affiliations:** Assistant Features Editor, Plant Physiology, American Society of Plant Biologists, Rockville, MD, United States; Department of Biology, Stanford University, Stanford, CA, United States; Department of Biology, Howard Hughes Medical Institute, Stanford, CA, United States

Salinity stress is a significant environmental stress that threatens plant growth. Soil salinization impacts over 6% of total land area and over 20% of all irrigated land (Munns and Tester 2008). Climate change exacerbates soil degradation through sea-level rise, shifting precipitation regimes, and elevated evapotranspiration rates. Consequently, it is estimated that half of the arable land will be lost due to increased salinity by 2050 (Jamil et al 2011). While most plants, including most crop species, are highly sensitive to salt stress, a small proportion (∼1%) of angiosperms, halophytes, are naturally adapted to thrive in saline soils containing over 200 mM NaCl (Rozema and Flowers 2008). Over the past millions of years, these plants have evolved diverse strategies to live with salt, one of which is to develop salt glands, as seen in the recretohalophytes, which are halophytes that secrete excessive ions through salt excretory structures on their leaves (Meng et al 2018).

Salt glands are specialized multicellular epidermal structures that facilitate the excretion of excess salt ions from leaves in recretohalophytes. While the morphological and anatomical development of salt glands in multiple recretohalophytes has been well characterized (Dassanayake and Larkin 2017), the molecular mechanisms of salt gland development and function remain elusive. With the availability of a chromosome-level genome assembly (Yuan et al 2022), a highly efficient tissue culture-based *Agrobacterium*-mediated transformation system (Yuan et al 2014), and a mutant library with an efficient screening method to identify salt gland mutants (Yuan et al 2015), *Limonium bicolor* (sea lavender) has become the model for investigating the development and salt secretion mechanism of multicellular salt glands. Consequently, this model system has facilitated the elucidation of numerous regulators contributing to salt gland development and salt secretion. Recently in *Plant Physiology*, Zhou et al (2026) reported the identification of 2 novel regulators, *LbWOX5* and *LbCUL1*, thereby expanding the known regulatory network for salt gland development and salt tolerance in *L. bicolor*.

As WUSCHEL-related homeobox (WOX) transcription factors are well-established regulators of plant development and abiotic stress responses, the authors first characterized the *LbWOX* gene family in *L. bicolor*. A total of 17 *LbWOX* members were identified, 8 of which (*LbWOX2*, *4*, *5*, *6*, *7*, *9*, *13*, and *14*) exhibited high expression levels during early salt gland morphogenesis. Functional analysis through individual gene silencing revealed that while none of these *LbWOX* genes affect leaf size, 5 members (*LbWOX2*, *4*, *5*, *6*, and *9*) appear to be involved in salt gland development. Notably, *LbWOX5* knockdown mutants displayed the most significant reduction in salt gland density. Further physiological characterization demonstrated that the salt secretion capacity of individual glands was markedly impaired in *LbWOX5*-silenced plants compared to the control plants under NaCl stress. Consequently, *LbWOX5* knockdown led to increased accumulation of Na^+^ and reactive oxygen species, including superoxide anions (O_2_^•−^) and hydrogen peroxide (H_2_O_2_), as well as elevated endogenous concentrations of stress indicators like proline and malondialdehyde. On the contrary, overexpression of *LbWOX5* in *L. bicolor* greatly increased salt gland density, improved the salt secretion capacity of individual glands, and reduced the accumulation of Na^+^, O_2_^•−^, and H_2_O_2_, as well as the stress biomarkers. Accordingly, *LbWOX5-*overexpressing plants produced more roots and leaves and higher biomass. More importantly, the authors found that *LbWOX5* is specifically expressed within salt glands during both the initiation and differentiation phases and localizes predominantly to the nucleus. These collectively indicated that *LbWOX5* acts as an essential regulator of salt gland development and function in *L. bicolor*.

Next, the authors identified *LbCUL1*, which encodes the central scaffold subunit of the Skp1–Cullin–F-box (SCF) E3 ubiquitin ligase complex, as a downstream target of LbWOX5. LbWOX5 directly binds to the promoter of *LbCUL1* and functions as a transcriptional activator in vitro. The expression of *LbCUL1* was highly reduced in *LbWOX5* knockdown lines, while its expression was magnified when *LbWOX5* was overexpressed. To determine whether *LbCUL1* is also involved in salt gland development, the authors generated both *LbCUL1*-silencing and *LbCUL1*-overexpressing plants. *LbCUL1* also functions as a positive regulator of salt gland development and function. Therefore, while *LbCUL1* knockdown plants accumulated more Na^+^ and suffered from more severe oxidative stress, *LbCUL1-*overexpressing mutants exhibited lower Na^+^ content and improved growth under NaCl treatment. Interestingly, when both *LbWOX5* and *LbCUL1* were silenced, the double mutants phenocopied either of the single mutants, and no additive effects on salt gland development and function were observed. These results demonstrated that *LbWOX5* directly regulates the transcription of *LbCUL1*, and they likely operate within the same regulatory pathway for salt gland development and function.

The past few years have seen the identification of many regulators of salt gland development and function in *L. bicolor*. To determine the connection between the known regulatory network and the LbWOX5-LbCUL1 module, the authors examined the expression of previously identified genes involved in salt gland development (*LbNAC55*, *LbSAD2*, and *LbVQ6*) and salt secretion (*LbSNAP33*, *LbNHX6*, and *LbSOS1*) in the *LbWOX5* and *LbCUL1* mutants. Compared to the control plants, all the tested genes increased their expression in *LbWOX5* overexpression plants and reduced their expression when either *LbWOX5* or *LbCUL1* or both were silenced, indicating that they likely function synergistically to regulate salt gland development and function.

In summary, the work by Zhou et al (2026) identified 2 new regulators, *LbWOX5* and *LbCUL1*, for salt gland development and salt tolerance in the recretohalophyte model *L. bicolor*, with the expression of *LbCUL1* modulated by *LbWOX5* during this process (Fig. 1). As LbWOX5 functions as a salt gland–specific transcription factor, it will be interesting to determine other targets of LbWOX5 in salt gland cells and examine their relations to salt gland development and function, as well as to investigate how LbWOX5 evolved to target and regulate salt gland cells. Further research will also be needed to examine whether the LbWOX5-LbCUL1 module also contributes to salt gland development and function in other recretohalophytes and to integrate individual regulatory modules that have been identified, such as LbWOX5-LbCUL1, into a comprehensive regulatory network for salt gland development and to understand how this network adapts to various environmental contexts. As trichomes and salt glands have been suggested to share a common ancestor (Yuan et al 2022), it will also be critical to determine what drives the distinct differentiation trajectories between trichomes and salt glands, as well as what confers the salt secretion capacity for salt glands. The answers to these questions will empower future research to evaluate the potential of engineering trichomes into salt-secreting glands in stress-sensitive plants, like crops, for improved stress resilience.

**Figure 1:**
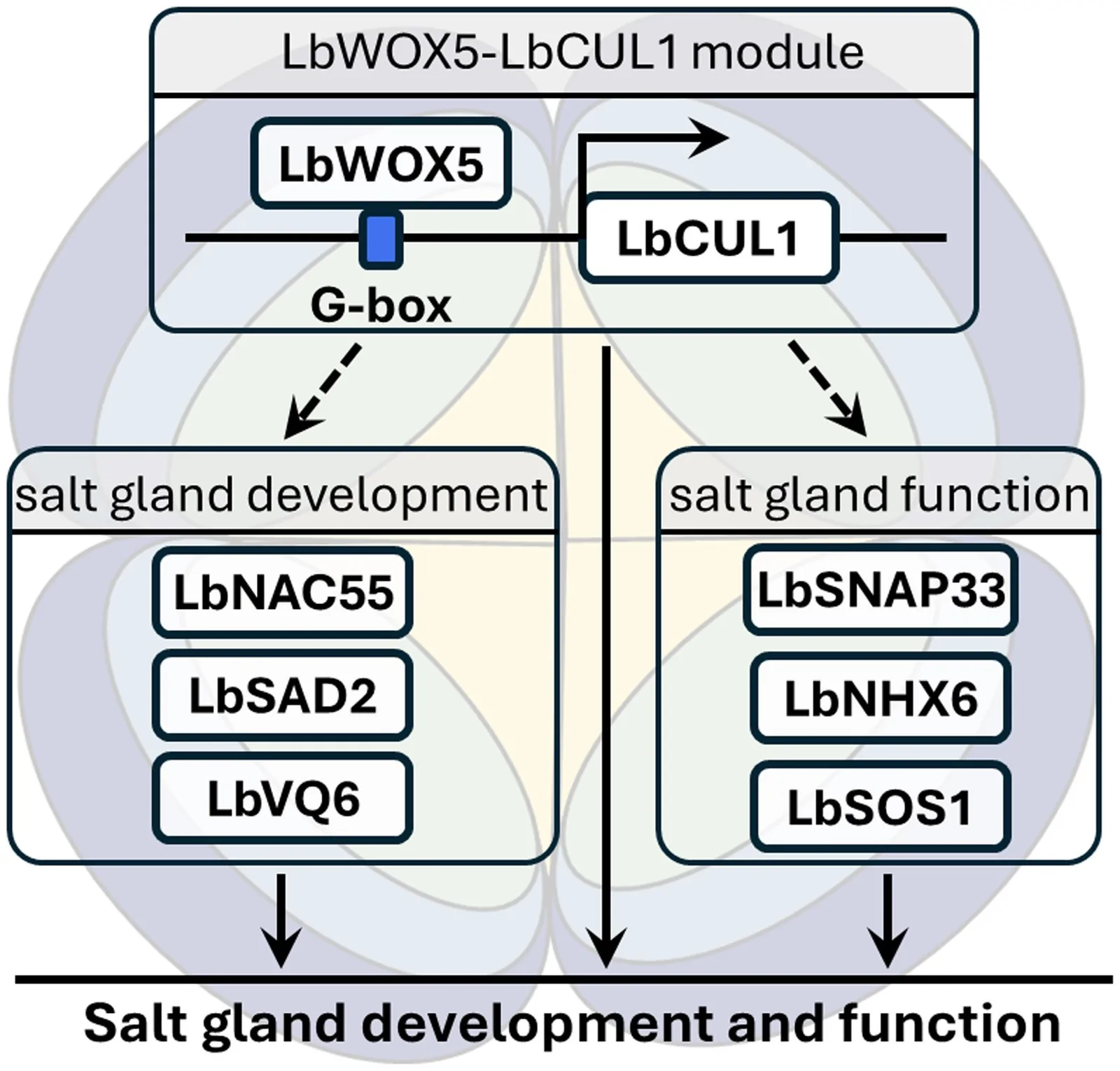
Regulation of salt gland development by the LbWOX5-LbCUL1 module in *Limonium bicolor*. CUL1, Cullin1; NHX6, Na^+^/H^+^ antiporter 6; SAD2, sensitive to ABA and drought 2; SNAP33, soluble N-ethylmaleimide-sensitive factor adaptor protein 33; SOS1, salt overly sensitive 1; VQ6, valine-glutamine motif-containing protein 6; WOX5, WUSCHEL-related homeobox 5.

**Recent related articles in *Plant Physiology*:**

- Zhao et al (2024) generated a single-cell atlas for developing leaves in *L. bicolor* and identified the developmental trajectory of salt glands.
- Duan et al (2025) reported a T2T gap-free genome for model halophyte *Salicornia europaea* and investigated the role of aquaporins in salt stress tolerance in this species.

## Data Availability

No new data were generated or analyzed in support of this research.
